# Electrocardiographic left ventricular hypertrophy with strain pattern: prevalence, mechanisms and prognostic implications

**Published:** 2008-02

**Authors:** OS OGAH, OO Oladapo, AA Adebiyi, BL Salako, AO Falase, AK Adebayo, A Aje, DB Ojji

**Affiliations:** Department of Medicine, Federal Medical Centre, Idi-Aba, Abeokuta, Ogun State, Nigeria; Department of Medicine, University College Hospital Ibadan; Department of Medicine, University of Ibadan, Nigeria; Department of Medicine, University College Hospital Ibadan; Department of Medicine, University of Ibadan, Nigeria; Department of Medicine, University College Hospital Ibadan; Department of Medicine, University of Ibadan, Nigeria; Department of Medicine, University College Hospital Ibadan; Department of Medicine, University of Ibadan, Nigeria; Department of Medicine, Lagoon Hospital Lagos, Lagos State, Nigeria; Department of Accident and Emergency Medicine, University College Hospital Ibadan, Nigeria; Department of Medicine, University of Abuja, Federal Capital Territory, Abuja, Nigeria

## Abstract

**Background:**

Electrocardiographic left ventricular hypertrophy with strain pattern has been documented as a marker for left ventricular hypertrophy. Its presence on the ECG of hypertensive patients is associated with a poor prognosis. This review was undertaken to report the prevalence, mechanism and prognostic implications of this ECG abnormality.

**Materials and methods:**

We conducted a comprehensive search of electronic databases to identify studies relating to the title of this review. The search criteria were related to the title. Two of the reviewers independently screened the searches.

**Results:**

Results were described qualitatively. The data were not pooled because there were no randomised studies on the topic. The prevalence of ECG strain pattern ranged from 2.1 to 36%. The highest prevalence was reported before the era of good antihypertensive therapy. The sensitivity as a measure of left ventricular hypertrophy ranged from 3.8 to 50%, while the specificity was in the range of 89.8 to 100%.

Strain pattern was associated with adverse cardiovascular risk factors as well as increased all-cause and CV morbidity and mortality.

ST-segment depression and T-wave inversion on the ECG was recognised as the strongest marker of morbidity and mortality when ECG-LV H criteria were utilised for risk stratification in hypertensive subjects.

**Conclusion:**

Electrocardiographic strain pattern identifies cardiac patients at higher risk of cardiovascular-related as well as all-cause morbidity and mortality.

## Summary

Electrocardiographic (ECG) left ventricular hypertrophy (LVH) with strain pattern is said to be present when, apart from the voltage criterion for ECG-LVH, there is also a downsloping asymmetrical ST-segment depression with inverted asymmetric T wave ≥ 0.1 mV opposite the QRS axis in a resting ECG. The generally accepted definition of left ventricular strain is an ST-segment depression that is bowed upwards and slopes down into an inverted asymmetrical T wave.^1^

The typical ECG strain pattern is a well-recognised marker of the presence of anatomical LVH and has been associated with an adverse prognosis in a variety of clinical conditions. ST-segment depression and T-wave inversion on the ECG is recognised as the strongest marker of morbidity and mortality when ECGLVH criteria have been utilised for risk stratification. Although the ECG strain pattern may also reflect the presence of underlying coronary artery disease, the strong association between strain on the ECG and increased LV mass has been shown to be independent of the presence of coronary artery disease.

We embarked on this review to define the documented prevalence, sensitivity and specificity of ECG-LVH and strain pattern relative to left ventricular structure and function, and the mechanisms and prognostic implications.

## Materials and methods

We systematically searched Medline from 1 January 1960 to December 2006. Other databases such as EMBASE, Cochrane Library, Web of Science were also searched for additional information.

A comprehensive search was developed based on the following search items: ‘electrocardiography’, ‘electrocardiographic (ECG) strain pattern’, ‘strain pattern’, ‘electrocardiographic ST-T changes’, ‘repolarisation abnormalities’ and ‘electrocardiographic left ventricular hypertrophy’. A review of the reference lists of the identified papers was conducted to identify relevant previously published articles. All the relevant papers were categorised and tabulated for descriptive analysis. Two of the authors independently screened the searches for relevant studies. Full manuscripts of these were retrieved. Studies of any design were included.

For the purpose of this work, ECG strain pattern was defined as the presence of LVH determined by voltage criteria, in addition to a downsloping asymmetrical ST-segment depression with inverted asymmetric T wave ≥ 0.1 mV opposite the QRS axis in a resting ECG.

One of the authors (OSO) extracted all the data using a standard proforma. These were checked for completeness and accuracy by another reviewer (AKA). Data were collected on prevalence of strain pattern, sensitivity and specificity, relationship between LV strain pattern and cardiac structure and function, and association between the abnormality and cardiovascular risk factors. Other data collected included mechanism of strain pattern and prognostic implications.

All the articles extracted were observational studies. There were no randomised controlled trials, hence we did not carry out further analysis on the studies.

## Results

**Fig. 1.**
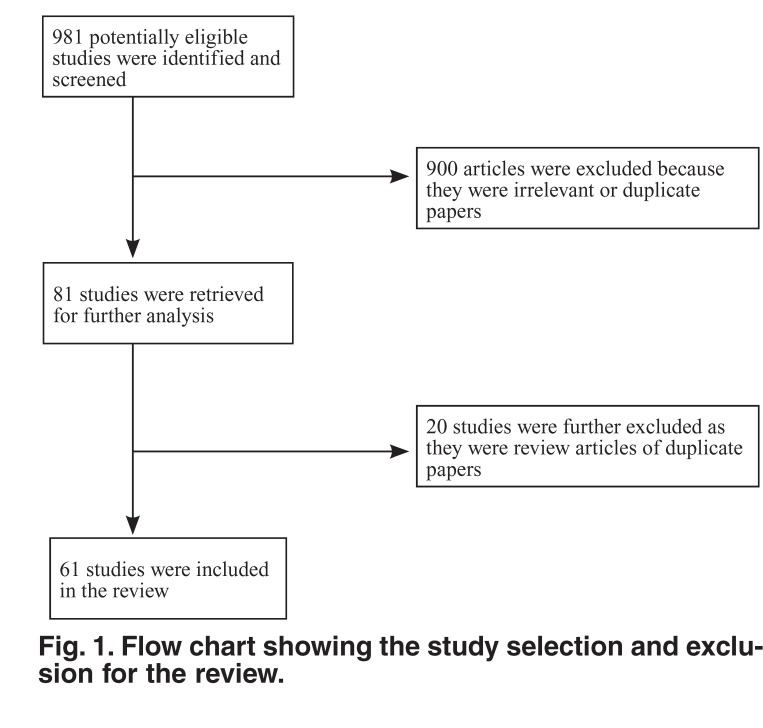
Flow chart showing the study selection and exclusion for the review.

Nine hundred and eighty-one studies were identified in our search and 61 were selected and retrieved for review. The rest were excluded because they were not relevant to the study, they were review articles, or were duplicate papers (Fig. 1).

For prevalence of ECG strain pattern, 16 studies were identified. 1-16 The prevalence ranged from 2.1 to 36%. The lowest prevalence was reported by Hsieh *et al.*,^3^ whereas the highest was found in 1960 by Simpson *et al.*^16^ (Table 1).

**Table 1.** Prevalence Of Electrocardiographic Strain Pattern

*No*	*Author*	*No of subjects*	*Year of publication*	*Prevalence (%)*
1	Okin *et al.*^2^	8696	2006	10.6
2	Hsieh *et al.*^3^	46, 950	2005	2.1
3	Okin *et al.*^5^	2193	2004	11.1
4	Opadijo *et al.*^6^	300	2003	16.7
5	Dahlof *et al.*^30^	524	2002	13 (men), 22 (women)
6	Okin *et al.*^8^	1595	2002	ST depression of any magnitude was present in 29.5% and only 0.1% had ≥ 100 μV (0.1 mV) of ST depression
7	Sundstrom *et al.*^1^	475	2001	11.1 (elderly men)
8	Okin *et al.*^31^	886	2001	15
9	Huston *et al.*^10^	766	1999	13.2
10	Verdecchia *et al.*^32^	1717	1998	6.4
11	Schillaci *et al.*^12^	923	1994	16
12	Dunn *et al.*^13^	3783	1990	12.8
13	HDFP		1979	3.5
14	Kannel *et al.*^14^	5581	1969	3.4
15	Sokolow *et al.*^15^	439	1961	19
16	Simpson *et al.*^16^	203	1960	36

HDFP 5 Hypertension Detection and Follow-up programme.

The sensitivity of ECG strain pattern ranged from 3.8 to 20% in the seven articles reviewed,^12^,^17^-^19^ while the specificity ranged from 89.8 to 100% (Table 2).

**Table 2.** Sensitivity And Specificity Of ECG Strain Pattern

*No*	*Author*	*Year of publication*	*Sensitivity (%)*	*Specificity (%)*	*Accuracy (%)*
1	Verdecchia^17^	2003	12.3	97.5	NA
2	Verdecchia^18^	2000	3.8	97.9	NA
3	Schillaci^12^	1994	16	89.8	NA
5	Fragola^19^	1993	20	100	NA
6	Vijan^33^	1991	15	100	44
7	Reicheck^34^	1981	50	93	80

NA 5 not available

We identified nine studies on the relationship between ECG strain pattern and LV structure and function.^9^,^20^-^27^ Most determined the association with LV systolic function, while two examined the relationship with echocardiographically determined LV diastolic function.^24^,^26^ Five studies reported on the impact of ECG strain pattern on LV geometry.^8^,^9^,^22^,^24^,^27^

ECG strain pattern was associated with poorer LV systolic function and abnormal LV geometry, particularly eccentric LVH. No relationship was found with LV diastolic function. Recently, ECG strain pattern has been shown to be associated with inappropriate left ventricular hypertrophy.^25^

Two studies reported on the association between ECG strain pattern and coronary circulation.^28^,^29^ This repolarisation abnormality was found to be associated with increased risk of coronary artery disease.

We extracted and reviewed one study on ECG strain pattern and LV wall motion.^35^ LV wall motion abnormalities were commoner in subjects with this ECG abnormality.

Two studies were identified on ECG strain pattern and the risk of sudden death.^28^,^36^ Sudden death occurred in 6.4% of subjects with strain pattern compared with 1.6% in individuals with ECG-LVH based on voltage criteria alone.

Three studies were identified on ECG strain pattern and the risk of cerebrovascular accident.^37^-^39^ The presence of ECG strain pattern was associated with excess risk for stroke.

Two studies were identified on ECG strain pattern and CV risk factors.^6^,^40^ Individuals with the abnormality were shown to have higher CV risk factors such as higher blood glucose and lipid levels. We found two studies on the mechanism of ECG strain pattern^41^,^42^ that tried to explain the underlying cause of LV strain pattern.

Thirteen studies were reviewed on the prognostic implications of ECG strain pattern.^1^,^2^,^4^,^5^,^9^,^13^-^15^,^17^,^29^,^32^,^37^,^39^,^43^-^49^ An adverse prognosis was associated with ECG strain pattern in the Framingham Heart study, Copenhagen City Heart study, Strong Heart study, LIFE study and at the Glasgow Blood Pressure Clinic (Table 3).

**Table 3.** Predictive Value Of ECG Strain Pattern

*No*	*Author*	*Year*	*Population*	*Predictive value*
1	Okin^2^	2006	Hypertensive subjects	ECG repolarisation abnormality was identified as a strong and significant predictor of incident congestive heart failure (HR = 1.80, 95% CI = 1.30−2.48) and congestive heart failure related death.
2	Okin^5^	2004	Type 2 diabetic American Indians	ST-depression predicted CV and all cause mortality after adjusting for age, gender, and other risk factors. HR = 3.68, 95% CI = 1.70−7.96 for CV mortality and HR = 2.36, 95% CI = 1.38−4.02 for all-cause mortality.
3	Aronow^46^, ^47^	1991/1998	Elderly subjects	ECG strain pattern (a component of Romhilt-Estes score) was associated with new-onset heart failure in elderly people
4	Okin^4^	2004	American Indians	Echocardiographic LVH and electrocardiographic ST depression was predictive of CV mortality (χ^2^ = 19.7, *p* = 0.01). The presence of both abnormalities was associated with 6.3-fold increased risk of CV death (95% CI = 2.8−14.2) and a 4.6-fold increased risk of all-cause mortality (95% CI = 2.5−8.5)
5	Pope^50^	2004	Emergency room patients	Strain pattern was associated with patients presenting at the emergency room with acute coronary syndrome.
6	Okin^37^	2004	Hypertensive subjects	ECG strain was shown to be a significant predictor of cardiovascular death (HR = 1.53, 95% CI = 1.18−2.00), myocardial infarction (HR = 1.55, 95% CI = 1.16−2.06) and composite endpoint (HR = 1.33, 95% CI = 1.11−1.59) after adjusting for traditional risk factors
7	Larsen^43^	2002	Copenhagen City Heart study cohort	Strain pattern at baseline was predictive of cardiac event. Did not find association between ECG strain pattern with congestive heart failure outcome
8	Sundstrom^1^	2001	Elderly men	ECG strain pattern was not a significant univariate predictor of all-cause or CV mortality
9	Verdecchia^32^	1998	Hypertensive Caucasians	Subjects with ECG strain pattern at baseline had a > 2-fold increased risk of new CV events and a 4.6-fold increased risk of CV mortality after adjusting for age, diabetes, previous CV events, smoking status and blood pressure.
10	Levy^45^	1994	Framingham cohort	ECG strain pattern was associated with age-adjusted risks of CV events of 5.8 (95% CI = 3.55−9.62) in men and 2.47 (95% CI = 1.38−4.42) in women.
11	Kannel^49^	1983	Framingham cohort	Presence of ECG strain pattern was associated with a more than 7-fold increased risk of developing heart failure after adjusting for confounders.
12	Kannel^48^	1970	Framingham cohort	Subjects with strain pattern had a > 3-fold increased risk of developing coronary artery disease after adjusting for age, gender and blood pressure
13	Sokolow and Perloff^15^	1961	Hypertensive subjects	Hypertensive subjects with strain pattern had an increased risk of mortality

## Discussion

## Historical perspective

Rykert and Hepburn^51^ introduced the concept of left ventricular strain in 1935. Beach *et al.*^44^ suggested that the repolarisation abnormalities of LVH without coronary artery disease could often be distinguished by the presence of one or more of the following: depression of the J-point, asymmetry of the T wave with rapid return to baseline, terminal positivity of the T wave (overshoot), T-wave inversion in lead V6 greater than 0.3 mV, and T-wave inversion greater in lead V6 than in V4.

It was Carter and Estes^52^ who in 1964 first showed that electrocardiographic evidence of left ventricular strain (which they defined as ‘asymmetric ST depression and T-wave inversion in the anterolateral leads, 1, AVL, V5 and/or V6’) was strongly associated with autopsy heart weight.

In 1982, Devereux *et al.*^27^ demonstrated that this ECG repolarisation abnormality was 95% specific for left ventricular hypertrophy in the absence of digitalis therapy. Okin *et al.*8 were the first to quantify the degree of ST depression in the ECG strain pattern and used this to predict mortality. The same group also went further to demonstrate that increasing ST depression in the lateral leads was associated with increasing LV mass and increased prevalence of anatomical LVH. Recently, they showed that ECG strain pattern was associated with incident congestive heart failure^2^ and inappropriate LVH.^25^

## Prevalence

ECG strain pattern occurs in both genders and in all races. It is said to be commoner in males, in black Africans and in people of black African descent.^31^,^53^ The prevalence of repolarisation abnormalities in hypertensive subjects reported in the literature ranged from 2.1% to as high as 36% (Table 1).^3^,^6^,^8^,^10^,^13^,^17^,^18^,^31^,^32^,^54^

ECG LVH detected by voltages alone was present in 48.8% of hypertensive Nigerians in Benin City in a study by Huston *et al.* of 766 subjects.^10^ Repolarisation abnormalities were present in 13.2% of the patients and in 1.0 to 2.8% of the normal controls.

Data from the Glasgow Blood Pressure Clinic showed that the prevalence of ECG-LVH was 34.5% in the group with high voltages alone and 12.8% in the groups with ST-T abnormalities. 13 In the Hypertension Detection and Follow-up Programme (HDFP), ECG-LVH diagnosed on the basis of voltage criteria only was present in 12.6% of the subjects while diagnosis requiring the presence of ST-T abnormalities resulted in 3.5% of the subjects having ECG-LVH.^55^ Van den Hoggen *et al.* reported a prevalence of 17 and 4% for voltage criteria and ST-T change criteria, respectively in the Netherlands.^54^ The LIFE study (Losartan Intervention For End-point study) and the Strong Heart study groups reported prevalences of 15 and 29.5%, respectively for electrocardiographic LVH with ST-T changes.^8^,^31^

## Variable patterns of ECG ST-T changes in hypertension

Classically, the ST-T changes in the ECG of patients with left ventricular hypertrophy are said to have a typical pattern of ST-segment depression and asymmetrical T-wave inversion. Many workers have described other patterns of ST-T changes in LVH.

In 1992, Huwez *et al.*^56^ reported a study of ST-T changes in the lateral leads of 24 patients with LVH documented by echocardiography. All had normal coronary arteries as determined by angiography. Sixteen of these patients had hypertension while eight had aortic valve disease. No patient was receiving digitalis preparations or had electrolyte imbalances, and none had had a previous myocardial infarction or ventricular conduction defects.

Typical electrocardiographic evidence of left ventricular strain was found in 63% of the patients (of these, 95% had asymmetrical T-wave inversion). Flat ST-segment depression, with or without T-wave inversion (symmetrical or asymmetrical) in the anterolateral leads was seen in 37% of subjects. Isolated T-wave changes without ST depression (symmetrical or asymmetrical) was found in 16% of the patients. They concluded that these variable ST-T changes could be produced by LVH without coronary artery disease.

Abnormalities of ventricular repolarisation have been well documented in normal and hypertensive black Africans and in people of black African origin. It is also well known that ventricular repolarisation in Africans takes diverse forms both in normal subjects and in the hypertensive population.^57^,^58^

## Relationship between ECG strain and cardiac structure and function

The presence of LV strain pattern on the resting 12-lead ECG has been shown to be associated with poorer cardiac systolic function. Badano *et al.*^59^ examined the electrocardiographic repolarisation changes and voltage criteria for LVH in relation to haemodynamic, echocardiographic and angiographic parameters. Their subjects consisted of 53 patients with aortic regurgitation and 36 patients with mitral regurgitation. They noted that patients with repolarisation abnormalities had a worse New York Heart Association (NYHA) functional class compared with those without repolarisation abnormalities. They also had greater LV dimensions, greater LV mass and higher LV enddiastolic pressure (LVEDP). LV strain was also associated with poor surgical outcome in their patients.

In another study by the same workers,^20^ ECG strain pattern was associated with higher peak meridian and circumferential stress and a more spherically shaped left ventricle. However, the ratio of the diastolic pressure–time index to the systolic pressure−time index, which is an index of myocardial oxygen supply-to-demand ratio, was similar in patients with or without strain patterns. They also documented that patients with ST-T changes were older than those without.

In a study by Radice *et al.*,^21^ impaired LV systolic function was shown both at rest and after exercise in patients who had ECG strain pattern. Roman *et al.*^22^ compared the LV systolic function of 41 subjects with LV strain and 54 without strain. Their subjects had severe and pure aortic regurgitation; none had evidence of coronary artery disease. All the subjects had echocardiography and radionuclide ciné-angiography. Greater LV internal dimensions and LV mass, higher end-systolic stress and lower endocardial fractional shortening were found in the group with LV strain pattern on their ECG. These differences were statistically significant. In a multiple regression analysis, LV mass and end-systolic stress were found to be independently related to the presence of repolarisation abnormalities. Yagi *et al.*^23^ documented similar findings.

Recently, we have shown that electrocardiographic LV strain pattern is associated with dilated left atrium, larger LV internal dimensions and greater absolute and indexed LV mass in hypertensive Nigerians.^24^

The relation of ECG strain pattern to LV diastolic function is less well documented. Recently, Palmieri *et al.* reported no difference in diastolic function parameters in hypertensive subjects with or without strain pattern.^26^ A similar finding was also reported from a study in native Africans.^24^ Some of the possible explanations include:^26^ (1) individuals with strain pattern may already have diastolic dysfunction that is not significantly worsened in the presence of strain pattern; (2) co-existing ischaemic heart disease in patients with strain pattern may modify both repolarisation and regional systolic−diastolic function; (3) deterioration in cardiac function is better characterised by indices of systolic function than by those of diastolic function.

## LV strain and LV geometry

The effect of ECG-LVH with strain pattern on LV geometry has been less well studied. Previous documentation on the relationship between strain and LV geometry has been inconsistent. Devereux *et al.*,^60^ Roman *et al.*,^22^ and Okin *et al.*^8^ reported a strong relationship between LV strain and eccentric hypertrophy as opposed to concentric hypertrophy.

In another study, concentric hypertrophy was found to be commoner in the hypertensive subjects with ECG features of repolarisation abnormality.^31^ The different populations in these studies could account for this. We also reported predominant eccentric geometry as opposed to concentric geometry in hypertensive Africans (Nigerians).^24^

Subjects with LV strain pattern are probably those who are at the end of the spectrum of hypertensive heart disease and who are likely to progress to the phase of congestive heart failure.

## ECG strain pattern and abnormalities of coronary circulation

A strong association has been documented between LV strain and obstructive coronary artery disease. The increased risk of subsequent myocardial infarction (MI) and coronary death in subjects with ECG strain pattern, noted in the Framingham study,^29^ has been confirmed by other workers.

In 1989, Pringle *et al.*^28^ reported 20 asymptomatic hypertensive patients with ECG strain pattern who had had coronary angiography. Eight of them were found to have one or more coronary artery lesions, which, on thallium scintigraphy, were associated with perfusion defects. One may therefore speculate that the association between strain pattern and increased levels of LV mass and wall stress, both of which increase myocardial oxygen demand, may be due, at least in part, to subendocardial ischaemia. The group concluded that hypertensive patients with LV strain pattern have significantly more episodes of exercise-induced ST-segment depression, reversible thallium perfusion abnormalities, significant non-sustained ventricular tachycardia on 24-hour ambulatory ECG, and significant coronary artery disease.

## ECG strain pattern and wall motion abnormality

Wall motion abnormalities have been documented to be commoner in hypertensives with ECG strain than those without this abnormality.

Palmieri *et al.*^35^ assessed the prevalence and the covariates of echocardiographic global and segmental left ventricular wall motion in 942 hypertensive patients with LVH in the Losartan Intervention For End-point reduction in hypertension (LIFE) echo substudy. Patients were separated into groups of those with normal wall motion and wall motion abnormalities. Compared with subjects with normal wall motion, those with wall motion abnormalities were mostly men and had a higher prevalence of ST-strain pattern. In a subanalysis restricted to patients with wall motion abnormalities, those with evident CV disease had a higher prevalence of ST-strain pattern than those with subclinical wall motion abnormalities, but other clinical ECG or echocardiography parameters were indistinguishable between the two groups.

## ECG strain pattern and the risk of sudden death

Hypertensive patients with ECG strain pattern have been shown to be at a higher risk of sudden death from arrhythmias compared with those without this pattern or with normal controls.

Pringle *et al.*^28^,^36^ studied the prevalence and significance of ventricular arrhythmias in 90 hypertensives with LVH and strain, using a Holter ECG and signal-averaging ECG, and programmed ventricular stimulation. They documented the presence of complex ventricular ectopic activities in 66% of the subjects and non-sustained ventricular tachycardia in 12%. This was similar to the findings of the Framingham Heart study groups where 6.4% of subjects with ECG strain pattern died suddenly compared with a figure of 1.6% for subjects who had had ECG-LVH detected by voltage criteria alone.

## LV strain and risk of cerebrovascular events in hypertension

The risk of cerebrovascular events in hypertension was higher in subjects with LV strain than in those without this repolarisation abnormality.^38^ Verdecchia *et al.* demonstrated that LVH detected by electrocardiography (using the Perugia score which has strain pattern as one of the components) conferred an increased risk for stroke (relative risk = 1.79; 95% CI = 1.07−2.68).^38^

## LV strain and cardiovascular risk factors

Studies have also shown that subjects with ECG strain pattern represent a subgroup with higher cardiovascular risk factors that must be aggressively followed up with multiple risk-factor intervention.

In a study of 300 hypertensive subjects categorised according to normal ECG, ECG-LVH by voltage criteria alone, and ECG strain pattern, it was observed that subjects with strain pattern were older, had significantly higher blood pressures, and higher body mass index (BMI), serum glucose and cholesterol values.^6^ In another study, Ichihara *et al.*^40^ studied 749 Japanese men who were selected according to their ECG features: normal ECG, LVH by voltage alone and LVH with abnormal ST-T segment. They demonstrated that Japanese men with ECG strain pattern had a higher BMI, and higher mean systemic blood pressure, blood glucose and high-density lipoprotein cholesterol levels than the normal ECG group or the group with ECG-LVH detected by voltage alone.

## Mechanisms of ECG strain pattern

The finding by many workers that the ECG strain pattern was associated with increased LV mass may suggest that myocardial hypertrophy may be a contributory factor. This is in agreement with the work by Thiry *et al.*^41^

Electrocardiographic strain pattern may also be due to subendocardial ischaemia, even in the absence of ischaemic heart disease. This is due to a hypertrophy-induced compensatory increase in the size of the coronary arteries, which are inadequate for the size of myocardial hypertrophy. This is supported by the fact that ECG strain pattern is related to increased wall stress-mass-heart rate product among patients who do not have coronary heart disease, evidence of ‘demand-side predisposition to myocardial ischaemia’.^42^ Moreover, it has been demonstrated that the ratio of the coronary artery lumenal area to regional LV mass can return to normal as a result of regression of LVH and/or repolarisation abnormality.^42^

## Prognostic implications of ECG strain pattern

LVH manifested by increased voltage as well as repolarisation abnormalities was found to have an adverse prognosis in the Framingham study.^45^,^61^ Within five years, 33% of men and 21% of women were dead. ECG strain was associated with ventricular ectopy and sudden death risk comparable to that of coronary heart disease and cardiac failure. It was also associated with a three- to 15-fold increase in the risk of cardiovascular events, with the greatest risk ratio for cardiac failure and stroke. In the Glasgow Blood Pressure Clinic,^13^ all-cause mortality expressed as death per 1 000 patient years was 27.6% for men with LVH only, and 59.6% for men with LVH and strain pattern. A similar trend was also obtained for women.

Some workers have looked at the predictive value of ECG strain pattern. The Framingham Heart study group^29^,^45^ reported that individuals with this pattern had a more than three-fold increased risk of developing coronary artery disease, after adjusting for gender, age and blood pressure. Larsen and coworkers^43^ of the Copenhagen City Heart study found that ECG strain pattern was predictive of cardiac events.

In the serial analysis of the ECG of 524 subjects (250 women and 274 men), Levy *et al.*^45^ showed that ECG strain was associated with age-adjusted risks of cardiovascular events after adjusting for the presence of baseline diabetes mellitus, serum cholesterol and cigarette smoking. They also demonstrated that the presence or absence of ECG strain pattern over time could be of value in risk stratification, with the presence of new strain patterns associated with increased risk, while resolution of strain was associated with a 50% reduction in the risk of cardiovascular events.

The study by Sokolow and Perloff^15^ showed that ECG strain pattern was associated with increased risk of cardiovascular-related mortality. In a study of 1 717 hypertensive patients, ECG strain pattern was associated with a more than two-fold increased risk of CV events and an adjusted 4.6-fold higher risk of CV mortality. The recent studies from the LIFE study group and the Strong Heart study have convincingly demonstrated the prognostic value of ECG strain pattern.

Okin *et al.*^4^,^9^ demonstrated that patients with strain pattern had a 2.26-fold higher risk of CV mortality than those without this abnormality. They also had a 2.16-fold increased risk of myocardial infarction, and a 1.85-fold increased risk of stroke. It was also a significant predictor of incident congestive heart failure.^2^ In the Strong Heart study,^5^,^37^,^39^ strain pattern was associated with a 6.3-fold increased risk of CV death (95% CI = 2.80−14.2) and a 4.6-fold increased risk of all-cause mortality (95% CI = 2.5−8.5)

## Conclusion

Electrocardiographic strain pattern identifies cardiac patients at higher risk of cardiovascular-related as well as all-cause morbidity and mortality. ECG strain pattern is associated with a higher cardiovascular risk, abnormal LV structure and function, incident heart failure, stroke and coronary artery disease.
